# Evaluating the learning curve of Minimally Invasive Chevron and Akin Osteotomy for correction of hallux valgus deformity: a systematic review

**DOI:** 10.1186/s12891-024-07940-x

**Published:** 2024-10-26

**Authors:** Luca Ramelli, Joon Ha, Shgufta Docter, Lucky Jeyaseelan, Mansur Halai, Sam Si-Hyeong Park

**Affiliations:** 1 Faculty of Medicine, Queen’s University, Kingston, Ontario Canada; 2 Barts Bone & Joint Health, Barts Health NHS Trust, London, UK; 3 Division of Orthopaedic Surgery, St. Michael’s Hospital, Toronto, Ontario Canada; 4 Division of Orthopaedic Surgery, University of Toronto, Toronto, Ontario Canada; 5 Division of Orthopaedic Surgery, Women’s College Hospital, 76 Grenville Street, Toronto, Ontario Canada M5S 1B2

**Keywords:** Hallux valgus, Bunions, Minimally invasive, Learning curve, Chevron, Akin

## Abstract

**Background:**

One procedure that has gained popularity in the surgical management of hallux valgus is the minimally invasive Chevron and Akin osteotomy (MICA). The purpose of this systematic review was to evaluate the learning curve associated with this technically demanding procedure.

**Methods:**

A search of the EMBASE and PubMed databases was performed to identify all clinical studies that assessed the learning curve associated with the MICA procedure. Studies where patients were not diagnosed with hallux valgus, did not undergo MICA, or did not report data on operation time, fluoroscopy exposure, or complications were excluded. A risk of bias assessment was conducted to assess the validity of the studies.

**Results:**

The initial literature search yielded 287 studies, and seven studies were included in the final analysis. A quantitative comparative analysis could not be performed as the included studies used different statistical methods to quantify the learning curve. Lewis et al. determined that after 38 operations, there was a decrease in operation time and fluoroscopy exposure (*p* < .001). Merc et al. found that it took 29 and 30 operations to reach a plateau for operation time and fluoroscopy exposure, respectively (*p* < .001). Palmanovich et al. found that it took 20 and 26 operations to reach a plateau for operation time and fluoroscopy exposure, respectively (*p* < .001). Toepfer and Strässle found there was a significant decrease in operation time and fluoroscopy exposure after the first 19 procedures in their series (*p* < .001). With respect to complications, one study found a significant difference after the 42nd operation (*p* = .007). However, the remaining studies found that complication rates did not significantly change with increased technical proficiency. All seven studies were deemed to have a moderate risk of bias.

**Conclusions:**

Surgeons can expect a learning curve of 20 to 40 operations before reaching technical proficiency with the MICA procedure. After the learning curve is achieved, surgeons can expect to see a significant decrease in both operation times and fluoroscopy exposure. No consistent significant difference was found in complications as one becomes more technically proficient with the procedure.

**Supplementary Information:**

The online version contains supplementary material available at 10.1186/s12891-024-07940-x.

## Introduction

Hallux valgus (HV) is a common foot deformity, with a prevalence of 23% in American adults aged 18 to 65 years old and 36% in American seniors aged 65 years and older [1]. Not only is HV common, but it also carries significant morbidity, with patients experiencing foot pain, impaired gait patterns, poor balance, and falls, especially in older adults [2]. Symptomatic HV can be operatively managed with either open or percutaneous techniques [3]. Minimally invasive surgery has been gaining widespread popularity and is becoming more accepted by patients and clinicians alike. More recently, there has been a growing interest in minimally invasive surgery for correction of HV, with percutaneous techniques offering benefits of smaller incisions, less pain, and quicker recovery, facilitating earlier rehabilitation and improved range of motion [4].

Multiple studies have shown that minimally invasive osteotomy procedures for correction of HV deformities have comparable long-term clinical outcomes with open procedures [5, 6]. The third generation minimally invasive Chevron and Akin osteotomy (MICA), originally described by Vernois and Redfern [7], is one technique for HV correction that has been shown to have good clinical and radiological outcomes [8, 9]. However, mastering the MICA procedure is recognized to be technically challenging. This can likely be attributed to the fact that minimally invasive procedures like MICA are primarily performed through tactile feedback and with complex hand-eye coordination demands, making it difficult for new learners to gain mastery of the procedure [10]. The learning curve and complication profile seen with minimally invasive techniques, coupled with the evidence that long-term outcomes are comparable between open and minimally invasive HV procedures, are just some of the many factors surgeons may consider when deciding to implement MICA into their practice.

## Methods

The purpose of this systematic review was to determine the learning curve associated with MICA hallux valgus correction to aid surgeons interested in incorporating this surgical procedure into their clinical practice. A systematic review of the literature was performed in accordance with the Preferred Reporting Items for Systematic Reviews and Meta-Analyses (PRISMA) guidelines [11]. A computerized search of the EMBASE and PubMed databases was performed from database inception to July 20, 2023, to identify studies involving the surgical correction of hallux valgus deformity with the third generation MICA procedure. Third generation MICA hallux valgus correction where patients undergo *both* a Chevron and Akin osteotomy was specifically selected so that comparison between studies could be performed. The search terms used included: “hallux valgus”, “bunions”, “MICA”, “minimally invasive”, “Chevron”, “Akin”, and “learning curve.” Search limits included English language studies only. Title and abstract screening and full text screening was performed in duplicate by two independent reviewers, and conflicts were resolved in collaboration with the senior author. Data extraction was performed using a standardized data extraction tool.

### Inclusion and exclusion criteria

The inclusion criteria were all clinical studies that examined the learning curve of the third generation MICA procedure for the treatment of HV. Clinical studies where patients were not diagnosed with HV, did not undergo MICA correction, or did not report data on at least one of operation time, fluoroscopy exposure, or complications, were excluded from the final analysis. Also, surgeries must have been performed in a consecutive fashion by a single surgeon for the learning curve to be assessed. Likewise, the outcome measures of interest were required to be reported in a stratified manner based on chronology to analyze the learning curve over time, either on a patient-by-patient basis, or by splitting groups of patients based on chronicity in the series.

### Risk of bias assessment

A risk of bias assessment was conducted in duplicate using the Cochrane Robins-I tool to assess the internal validity of the clinical studies used in the final analysis. Each of the studies were analyzed in the different domains outlined in the tool and identified as having a low, moderate, or high risk of bias in each domain. An overall risk of bias assessment was assigned to each study based on the internal validity of the study in each of the domains.

## Results

**Fig. 1:**
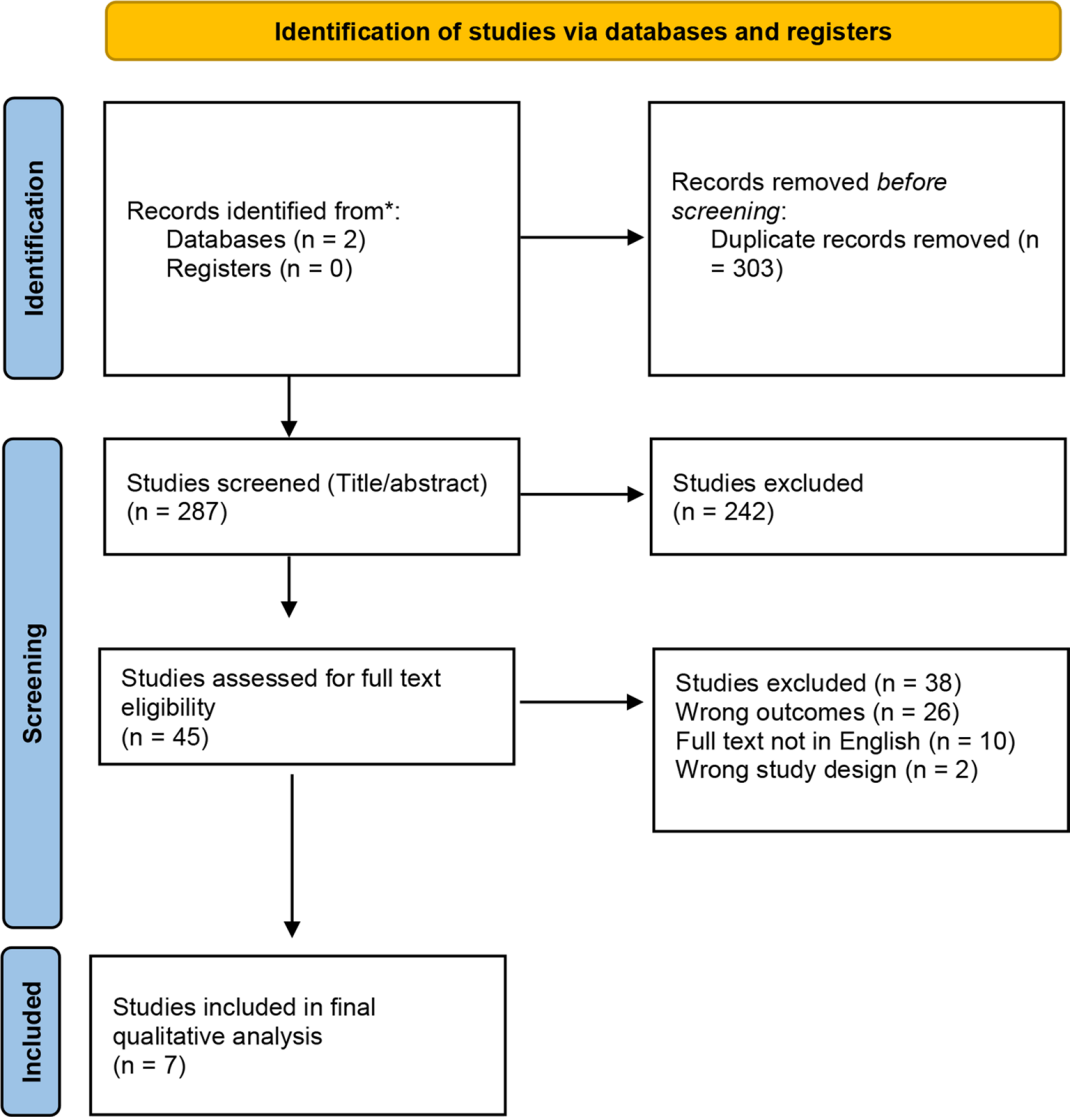
PRISMA [11] diagram displaying the results of the literature search conducted

The initial literature search resulted in 287 studies. After screening for the inclusion and exclusion criteria, a total of seven studies were identified (Fig. 1) [12–18]. Of the seven studies, four were prospective case series [12, 13, 16, 18] and three were retrospective case series [14, 15, 17]. The characteristics of the included studies are illustrated in Table 1.

**Table 1:** Study characteristics

Author (Year)	Country	Study Design	Mean Age ± SD/range (years)	Number of operations
Jowett and Bedi (2017) [12]	Australia	Prospective case series	55 ± 13.3	106 (over 26 months)
Karry et al. (2015) [13]	Hong Kong	Prospective case series	52.5 ± 10.4	13 (over 23 months)
Lewis et al. (2023) [14]	UK	Retrospective case series	57.5 ± 13.4	58 (over 16 months)
Merc et al. (2023) [15]	Slovenia	Retrospective case series	50 ± 15	100 (over 24 months)
Neufeld et al. (2021) [16]	USA	Prospective case series	56.8 (15–84)	94 (over 14 months)
Palmanovich et al. (2020) [17]	Israel	Retrospective case series	53 (17–81)	50 (over 36 months)
Toepfer and Strässle (2022) [18]	Switzerland	Prospective case series	57.4 (25–78)	50 (over 33 months)

Of the seven studies included in the final analysis, only two studies [14, 15] reported on all three of the outcomes of interest (operative time, fluoroscopy exposure, and complications). Three other studies reported on operation time [13, 17, 18], with two of those also reporting fluoroscopy exposure [17, 18]. Five of the seven studies reported on complications [12–16]. The reported complications were quite variable (please refer to the tables in the Supplementary files for a detailed breakdown of the complications). The complications reported by Jowett and Bedi included symptomatic hardware requiring removal, revision surgeries including osteotomy and EHL tendon lengthening, wound infection, periprosthetic fracture, medial exostosis removal, delayed union, under/overcorrection, recurrence, scar sensitivity, and first metatarsal shortening [12]. Karry et al. reported complications related to symptomatic hardware and sutures requiring reoperation [13]. Lewis et al. reported the need for revision surgery due symptomatic hardware and medial exostosis removal [14]. The complications reported by Merc et al. included removal of symptomatic hardware, revision surgery for first metatarsal head malposition, first MTP joint stiffness, infection, and loss of fixation [15]. Finally, Neufeld et al. reported on hardware complications requiring removal, infection, neurologic injury, medial prominence irritation, loss of reduction/hardware backout, scar sensitivity, and intraoperative first metatarsal fracture in their series of patients [16].

Four studies used a statistical method to determine the learning curve [14, 15, 17, 18] and reported patient data as two separate groups: a “learning phase” group and a “plateau phase” group. Two other studies split their reporting data with the first half of patients in the series as one group (labelled “A”), and the second half of patients in their series as another group (labelled “B”) [12, 16]. For the purposes of harmonizing the different language used between studies, the group A from the two studies will be referred to as the “learning phase” group and the group B as the “plateau phase” group. A summary of the outcome data collected by each study is displayed in Tables 2, 3 and 4. Data could not be pooled between studies due to different statistical analyses used to assess learning curves and a lack of primary data available. Thus, a quantitative comparative analysis could not be performed.

Lewis et al. determined that 38 operations were required to overcome the MICA learning curve [14], as there was a statistically significant decrease in mean operation time (68.7 min versus 53 min, *p* < .001) and mean number of fluoroscopy pictures (121.6 pictures versus 102.9 pictures, *p* = .01) from the first 38 procedures compared to the last 20 procedures in the series [14]. Merc et al. found that it took 29 operations to reach a plateau for operation time (median of 60 versus 40 min, *p* < .001); however, the plateau for fluoroscopy exposure was not reached until after 30 operations were performed (median of 92 pictures versus 69 pictures, *p* < .001) [15]. Palmanovich et al. found that it took 20 operations to reach a plateau for operation time (84.1 min versus 45 min, *p* < .001); however, the plateau for fluoroscopy exposure was not reached until after 26 operations were performed (164 pictures versus 76.1 pictures, *p* < .001) [17]. In the study conducted by Toepfer and Strässle, there was a statistically significant decrease in mean operation time (57.3 min versus 43.1 min, *p* < .001) and mean number of fluoroscopy pictures (166.5 pictures versus 113.1 pictures, *p* < .001) from the first 19 procedures compared to the last 31 procedures in the series [18]. With respect to complication rates, the studies by Lewis et al. [14] and Neufeld et al. [16] determined that complications did not change significantly with increased technical proficiency and more cases performed in their respective series (*p* = .356 [14] and *p* = .289 [16], respectively). In the study by Jowett and Bedi [12], there was a slightly significant difference between the learning phase and plateau phase (51% versus 32%, *p* = .047) when all complications were considered [12]. However, when only symptomatic complications requiring reoperation were examined, the authors found no statistical difference between the learning and plateau phase (26% versus 15%, *p* = .155) [12]. Jowett and Bedi also reported on the HV recurrence rate and found no significant difference between the learning and plateau phase (13% versus 9%, *p* = .509) [12]. Merc et al., however, determined that after the first 42 operations in their study, there was a significant difference in complication rates (36% versus 12%, *p* = .007) [15].

Lastly, the study conducted by Karry et al. had only 13 operations performed, with a mean operative time of 102.5 min and a reoperation rate of 31% [13]. The learning curve was unable to be determined from this study as not enough operations were performed to reach a plateau in technical proficiency.

**Table 2:** Summary of operation times before and after technical proficiency was reached

Study	*N* of operations before technical proficiency	Mean/median operation time (min) before technical proficiency	Mean/median operation time (min) after technical proficiency	*P* value
Jowett and Bedi (2017) [12]	-	-	-	-
Karry et al. (2015) [13]	-	102.5 ± 23.9	-	-
Lewis et al. (2023) [14]	38	68.7 ± 18	53 ± 9.2	< 0.001
Merc et al. (2023) [15]	29	60 (median)	40 (median)	< 0.001
Neufeld et al. (2021) [16]	-	-	-	-
Palmanovich et al. (2020) [17]	20	84.1 ± 40.2	45 ± 6	< 0.001
Toepfer and Strässle (2022) [18]	19	57.3 ± 12.1	43.1 ± 8.1	< 0.001

**Table 3:** Summary of fluoroscopy exposure before and after technical proficiency was reached

Study	*N* of operations before technical proficiency	Mean/median fluoroscopy exposure (*N* of pictures) before technical proficiency	Mean/median fluoroscopy exposure (*N* of pictures) after technical proficiency	*P* value
Jowett and Bedi (2017) [12]	-	-	-	-
Karry et al. (2015) [13]	-	-	-	-
Lewis et al. (2023) [14]	38	121.6 + 21.6	102.9 + 29.2	0.01
Merc et al. (2023) [15]	30	92 (median)	69 (median)	< 0.001
Neufeld et al. (2021) [16]	-	-	-	-
Palmanovich et al. (2020) [17]	26	164 + 52.6	76.1 + 26.7	< 0.001
Toepfer and Strässle (2022) [18]	19	166.5 + 30.4	113.1 + 29.5	< 0.001

**Table 4:** Summary of complications before and after technical proficiency was reached

Study	*N* of operations before technical proficiency	Complications before technical proficiency (first half of patients in series)	Complications after technical proficiency (second half of patients in series)	*P* value
Jowett and Bedi (2017) [12]	-	51% (Operations 1–53)	32% (Operations 54–106)	0.047
Karry et al. (2015) [13]	-	31%*	-	-
Lewis et al. (2023) [14]	38	5%	15%	0.356
Merc et al. (2023) [15]	29 for operation time, 30 for fluoroscopy exposure	36% (Operations 1–42)	12% (Operations 43–100)	0.007
Neufeld et al. (2021) [16]	-	15% (Operations 1–47)	8% (Operations 48–94)	0.289
Palmanovich et al. (2020) [17]	20 for operation time, 26 for fluoroscopy exposure	-	-	-
Toepfer and Strässle (2022) [18]	19	-	-	-

*Karry et al. reported total complication rates across the 13 patients in their series

## Risk of Bias Assessment

**Fig. 2:**
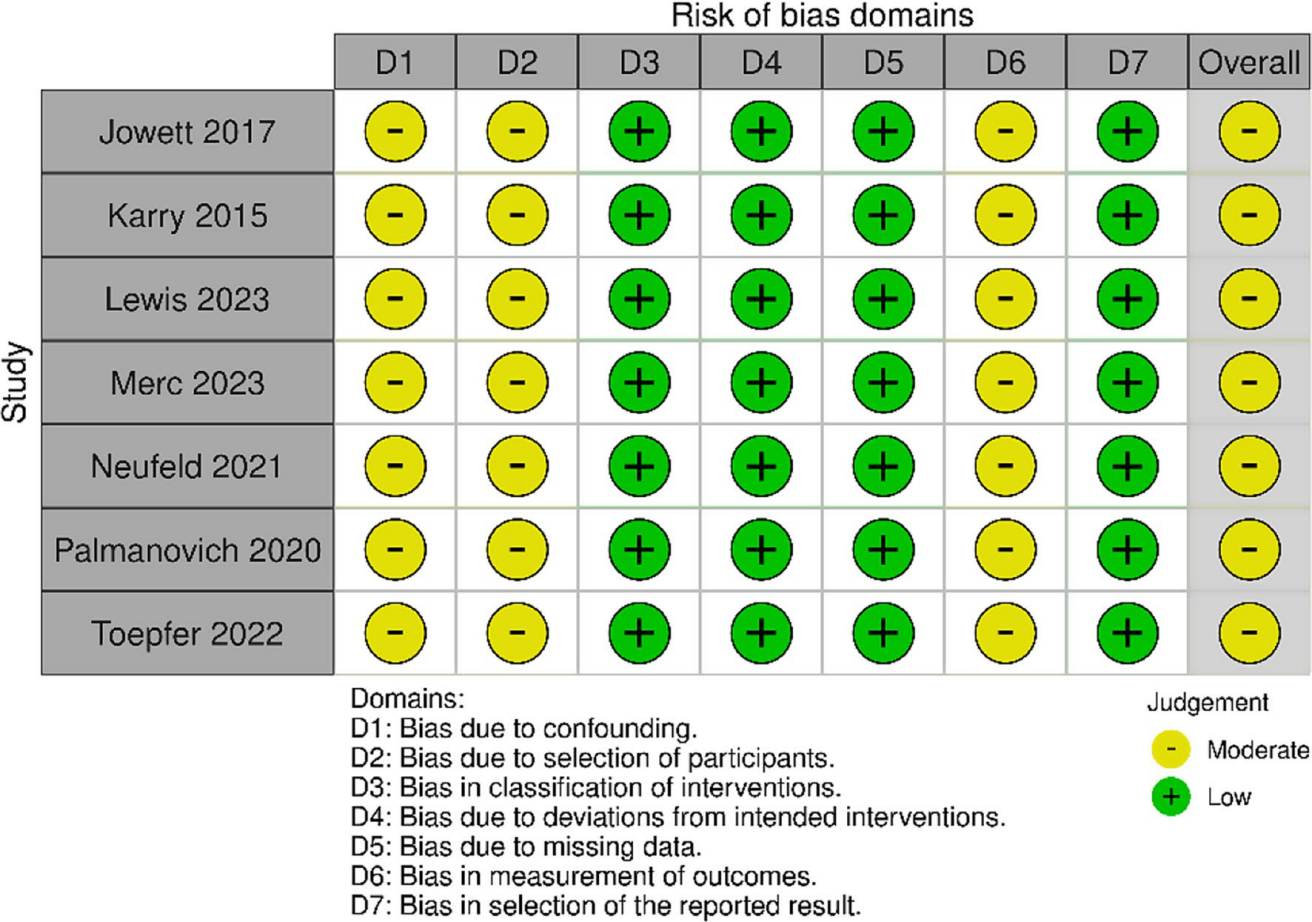
Outcome of risk of bias assessment [19]

The results of the risk of bias assessment are displayed in Fig. 2. All seven studies were deemed to have some concern of bias (“moderate”) [12–18]. All of the studies were deemed to have at least a moderate risk of bias in the domain of confounding as they were non-randomized studies. Additionally, all studies were deemed to have risk of misclassification bias as the design of the studies did not allow for blinding of outcome assessors. On the other hand, all studies were deemed to have a low risk of bias in classification of interventions, as the surgical technique performed for each patient was documented and explicitly defined.

## Discussion

This systematic review examined the learning curve associated with the third generation MICA procedure for the correction of HV deformity. Based on the findings, surgeons who are interested in incorporating the MICA procedure into their clinical practice can expect a learning curve of at least 20 operations and likely no more than 40 operations before reaching technical proficiency. Once the learning curve is overcome, surgeons can expect to see a significant decrease in both operation times and fluoroscopy exposure.

Analysis of the studies included in this systematic review revealed that different methods were used to determine and calculate the MICA learning curve – hence limiting the ability to perform a quantitative comparative analysis. Lewis et al. measured the learning curve based solely on operation time [14]. The operation time was plotted against case number and the plot was fitted with a smoothed cubic spline with four knots of freedom [14]. Straight lines were then fitted to the curve to define the learning period and the inflection point to the asymptote or plateau phase, which was found to be after case 38 [14]. In the study conducted by Merc et al., a dissociation curve was fit to each data set of operation time and fluoroscopy exposure, and when solving the derivate of the equation for each curve using the calculated linear slope, the result was 29 and 30 respectively [15]. In the study conducted by Palmanovich et al., a two-tailed Spearman’s rank-order correlation test was used to determine the relationship between the surgeon’s experience and the fluoroscopy exposure or the operation time [17]. By excluding the first cases in a manual stepwise manner and repeating the same analysis, they found that from patient 27 to 46, the accumulative experience did not lead to less intraoperative fluoroscopy exposure (*rs = −* 0.38, *p* = .07) [17]. Likewise, starting at the 21st patient, the surgeon’s accumulative experience did not lead to shorter operations (for patients 21 to 46, *rs = −* 0.31, *p* = .1) [17]. Through this analysis, Palmanovich et al. determined that it took 21 to 26 operations to overcome the MICA learning curve [17]. Lastly, Toepfer and Strässle plotted their patients’ operation time and fluoroscopy exposure on two separate scatter plots and used those plots to qualitatively estimate approximately when there was a consistent downward trend in operative time and fluoroscopy exposure [18]. Using the plot generated by Toepfer and Strässle, the learning curve was estimated to be overcome after 19 cases [18]. In each of these four studies, there was a statistically significant difference (*p* between 0.01 and < 0.001) in both operative times and fluoroscopy exposure when comparing patient data prior to the calculated learning curve and after the learning curve was reached – thereby confirming there is indeed a learning curve present no matter how it is calculated [14, 15, 17, 18]. The study by Karry et al. further supported the notion that at least 20 operations can be expected to reach technical proficiency. In their study, they found a mean operative time of 102.5 min and a reoperation rate of 31% for the first 13 MICA procedures performed [13]. These findings were comparable to the learning phases reported in studies presented in this review.

The learning curve being between 20 and 40 procedures for the MICA technique is consistent with other minimally invasive procedures described in the orthopaedic literature. For example, one study on minimally invasive total hip arthroplasty found that 40 cases were required to overcome the learning curve [20]. Within the spine literature, the learning curve of minimally invasive spine surgeries for a variety of cervical-thoracic-lumbar procedures was found to be between 20 and 30, and up to 44 cases for transforaminal lumbar interbody fusions before seeing significant decreases in operative times and reoperation rates [21].

Within the foot and ankle literature, the learning curve of the MICA procedure was not much different when compared to open procedures. In a study of patients who underwent ankle, hindfoot, or midfoot procedures, it was shown that functional outcomes were worse for the first 27 patients operated on when compared to the following 30 patients in a series performed by a single surgeon [22]. As well, one study investigating the learning curve of the modified Lapidus procedure for HV correction found that technical proficiency was achieved after 23 cases [23]. Lastly, Seng et al. found a learning curve of 24 procedures for their case series of HV correction via open Scarf osteotomy [24]. Although the MICA procedure is thought to be a technically challenging procedure to perform, the findings of our systematic review have shown that the learning curve is comparable to many other procedures within orthopaedics.

Surgeons may be hesitant to adopt new surgical techniques because of the concern of worse clinical outcomes and increased complications, especially during the early learning phase. However, our review found no consistent difference in complications as a surgeon becomes more technically proficient with the MICA procedure. Furthermore, the rates of reoperation in the learning phase of the MICA procedure were comparable to open procedures. Coetzee et al. reported a reoperation rate of greater than 35% in the first 20 patients who underwent open Scarf osteotomy for HV correction [25]. This was comparable to the reoperation rates reported by Jowett and Bedi (26% in first 53 patients) [12] and Karry et al. (31% in first 13 patients) [13], and higher than those reported by Lewis et al. (5% in first 38 patients) [12] and Neufeld et al. (6% in first 47 patients) [16] for MICA HV correction. This finding is reassuring for surgeons interested in adopting the MICA procedure into their clinical practice.

The study conducted by Merc et al. found a significant difference in complications during the learning phase of the procedure compared to the plateau phase [15]. It is important to note that 77% of these complications were hardware-related and originated from suboptimal screw position, and the significant reduction in complication rates after case 42 was attributed to the surgeon’s refinement of the screw placement technique [15]. Otherwise, the lack of any difference in complications in the learning versus plateau phase illustrated in the other studies may be attributable to a variety of factors. Although the surgical time may decrease with repetition and more familiarity with the procedure, the steps and principles of the procedure remain unchanged. If the surgical technique itself does not change, the risk factors associated with the operation and the potential for complications requiring reoperation (e.g. malunion, non-union, and hardware irritation) will likely remain constant. Lastly, if the patient population undergoing the procedure remains the same, the complication rates do not change. Patients with comorbidities or complex medical histories will still be at risk of the same complications and reoperations despite the surgeon reaching technical proficiency.

In our current study, the primary dependent variables of improvement in operative time and fluoroscopy exposure were convenient surrogate measures of proficiency, but ultimately do not accurately reflect true surgical competence or patient outcomes. Unfortunately, the majority of studies included in our review did not report any patient reported outcome measures. Only one study (Jowett and Bedi [12]) reported AOFAS scores that were divided by the learning phase and the plateau phase. The authors found that although there were improvements in the preoperative versus postoperative AOFAS scores for both groups, the degree of improvement was essentially the same between the learning and plateau phase [12]. Furthermore, the study by Jowett and Bedi showed that there was no difference in rates of recurrence of HV deformities. Future studies should factor in patient reported outcomes when assessing learning curves to provide a holistic perspective.

A review conducted by Bedi and Hickey identified several ways that surgeons can decrease the learning curve for minimally invasive orthopaedic procedures [26]. Several key points were identified, namely the importance of cadaveric training and the importance of implementing strategies to flatten the learning curve [26]. Cadaveric training is important for surgeons to become familiar with instruments and key parts of the procedure [24]. Next, identifying and selecting patients with easier to correct deformities (which in our experience are those with more moderate HV deformities) and patients with low intra-/post-operative complication risks when a surgeon is beginning to implement MICA into their practice may be beneficial to flattening the learning curve as the surgeon becomes more proficient with the technique [14, 26]. It is also important to reflect on cases performed and the measurement of outcomes so that surgeons can continually identify areas for improvement [26]. A surgeon who is committed to implementing MICA as part of their practice can use strategies such as the ones described above to potentially decrease their learning curve below the minimum of 20 cases that has been reported in this study. It is important to note that this review serves a guideline as to when a surgeon can likely begin to see improvements in their technical proficiency; however, we recognize that every surgeon is different.

## Strengths and limitations

The key strengths of this review include the comprehensive search and article screening process, as well as a clearly stated objective to answer a focused question of clinical relevance. This review is the first article that has synthesized all the learning curve data available in the literature specifically pertaining to the third generation MICA procedure in order to inform surgeons interested in adding the MICA technique to their armamentarium.

Overall, the relative novelty of the MICA procedure and the small number of studies specifically examining the learning curve of the MICA procedure are the main limitations when assessing the results of this systematic review. A lack of primary data in operative time, fluoroscopy exposure, and complications precluded pooling of data. Furthermore, studies could not be pooled because the inflection point of proficiency differed between studies, making between-group comparisons inappropriate. In addition, all studies included in this review were deemed to have some threat to internal validity, thereby affecting the quality of the evidence presented in this review. Ultimately, a larger volume analysis of multiple surgeons in diverse centres undertaking the same surgical technique would reinforce the validity of the findings of this systematic review.

## Conclusions

Although the MICA procedure is a challenging and technically demanding operation, surgeons can expect to see a significant improvement in operation times and fluoroscopy exposure between 20 and 40 cases. Surgeons should be reassured that the learning curve with respect to complications and reoperations appears to be consistent, particularly as the majority of studies suggest that an increase in the number of MICA procedures performed does not necessarily lead to decreased complication rates. The learning curve for the MICA procedure is comparable to the learning curve of other orthopaedic procedures, both open and minimally invasive.

## Electronic supplementary material

Below is the link to the electronic supplementary material.

Supplementary Material 1

## Data Availability

All data generated or analysed during this study are included in this published article and its supplementary information files.

## Abbreviations

HV: Hallux valgus
MICA: Minimally Invasive Chevron and Akin Osteotomy
PRISMA: Preferred Reporting Items for Systematic Reviews and Meta-Analyses
