# Supplementary material for: Evaluating the learning curve of Minimally Invasive Chevron and Akin Osteotomy for correction of hallux valgus deformity: a systematic review
Source: BMC Musculoskelet Disord. 2024 Oct 26;25:854. doi: 10.1186/s12891-024-07940-x (PMC11515154; doi:10.1186/s12891-024-07940-x)
Supplement: Supplementary file 1 — Supplementary Material 1 [file 12891_2024_7940_MOESM1_ESM.docx]

**APPENDIX**

Detailed summary of reported complications by each study.

| **Table 1. Jowett and Bedi (2017)^12^** | | |
| --- | --- | --- |
| **Complication** | **Number**  *(Learning Phase)* | **Number**  *(Plateau Phase)* |
| Symptomatic screws requiring removal | 10 | 6 |
| Medial exostosis removal | 1 | 0 |
| Revision osteotomy and bone graft | 1 | 0 |
| EHL tendon lengthening required after index surgery | 1 | 1 |
| Wound infection | 1 | 1 |
| Periprosthetic fracture | 0 | 1 |
| Delayed union *(asymptomatic)* | 2 | 1 |
| Under/overcorrection | 2 | 0 |
| Recurrence | 7 | 5 |
| Scar sensitivity | 2 | 0 |
| First metatarsal shortening >5 mm *(asymptomatic)* | 0 | 1 |

*Total number of study patients in *Learning Phase* = 53.

**Total number of study patients in *Plateau Phase* = 53.

| **Table 2. Karry et al (2015)^13^** | |
| --- | --- |
| **Complication** | **Number** |
| Removal of Akin screw due to impingement | 4 |
| Suture removal due to medial plication stitch impingement | 4 |

*Total number of study patients = 23.

| **Table 3. Lewis et al (2023)^14^** | |
| --- | --- |
| **Complication** | **Number** |
| Revision of medial exostosis | 4 |
| Removal of screw for soft tissue irritation | 1 |

*Total number of study patients = 58.

| **Table 4. Merc et al (2023)^15^** | |
| --- | --- |
| **Complication** | **Number** |
| Hardware removal (due to local soft tissue irritation) | 17 |
| Revision surgery for first metatarsal head malposition | 2 |
| First MTP joint stiffness | 1 |
| Infection | 1 |
| Loss of fixation | 1 |

*Total number of study patients = 100.

| **Table 5. Neufeld et al (2021)^16^** | |
| --- | --- |
| **Complication** | **Number** |
| Hardware removal | 3 |
| Neurologic injury (neurolysis, persistent paresthesia) | 3 |
| Infection | 2 |
| Persistent scar sensitivity | 1 |
| Intraoperative first metatarsal fracture | 4 |
| Loss of reduction/hardware backout | 3 |
| Medial prominence irritation | 4 |

*Total number of study patients = 94.
